# Differential Neuroplastic Changes in Fibromyalgia and Depression Indexed by Up-Regulation of Motor Cortex Inhibition and Disinhibition of the Descending Pain System: An Exploratory Study

**DOI:** 10.3389/fnhum.2019.00138

**Published:** 2019-04-25

**Authors:** Tiago Madeira Cardinal, Luciana Conceição Antunes, Aline Patricia Brietzke, Cristiane Schulz Parizotti, Fabiana Carvalho, Andressa De Souza, Iraci Lucena da Silva Torres, Felipe Fregni, Wolnei Caumo

**Affiliations:** ^1^Post-graduate Program in Medical Sciences, School of Medicine, Universidade Federal do Rio Grande do Sul, Porto Alegre, Brazil; ^2^Department of Nutrition, Health Science Center, Universidade Federal de Santa Catarina, Florianópolis, Brazil; ^3^Post-graduate Program in Health and Human Development, Universidade La Salle, Canoas, Brazil; ^4^Department of Pharmacology, Instituto de Ciências Básicas da Saúde, Universidade Federal do Rio Grande do Sul, Porto Alegre, Brazil; ^5^Department of Surgery, Pain, and Anesthesia, School of Medicine, Universidade Federal do Rio Grande do Sul, Porto Alegre, Brazil; ^6^Anesthesiologist, Pain and Palliative Care Service, Hospital de Clínicas de Porto Alegre, Laboratory of Pain and Neuromodulation, Universidade Federal do Rio Grande do Sul, Porto Alegre, Brazil

**Keywords:** fibromyalgia, depression, primary motor cortex, pain, CPM, BDNF

## Abstract

**Background:** Major depressive disorder (MDD) and fibromyalgia (FM) present overlapped symptoms. Although the connection between these two disorders has not been elucidated yet, the disruption of neuroplastic processes that mediate the equilibrium in the inhibitory systems stands out as a possible mechanism. Thus, the purpose of this cross-sectional exploratory study was: (i) to compare the motor cortex inhibition indexed by transcranial magnetic stimulation (TMS) measures [short intracortical inhibition (SICI) and intracortical facilitation (ICF)], as well as the function of descending pain modulatory systems (DPMS) among FM, MDD, and healthy subjects (HS); (ii) to compare SICI, ICF, and the role of DPMS evaluated by the change on Numerical Pain Scale (NPS) during the conditioned pain modulation test (CPM-test) between FM and MDD considering the BDNF-adjusted index; (iii) to assess the relationship between the role of DPMS and the BDNF-adjusted index, despite clinical diagnosis.

**Patients and Methods:** A cohort of 63 women, aged 18 to 75 years [FM (*n* = 18), MDD (*n* = 19), and HC (*n* = 29)].

**Results:** The MANCOVA analysis revealed that the mean of SICI was 53.40% larger in FM compared to MDD [1.03 (0.50) vs. 0.55 (0.43)] and 66.99% larger compared to HC [1.03 (0.50) vs. 0.34 (0.19)], respectively. The inhibitory potency of the DPMS assessed by the change on the NPS during CPM-test was 112.29 % lower in the FM compared to MDD [0.22 (1.37) vs. −0.87 (1.49)]. The mean of BDNF from FM compared to MDD was 35.70% higher [49.82 (16.31) vs. 14.12 (8.86)]. In FM, the Spearman’s coefficient between the change in the NPS during CPM-test with the SICI was Rho = −0.49, [confidence interval (CI) 95%; −0.78 to −0.03]. The BDNF-adjusted index was positively correlated with the disinhibition of the DPMS.

**Conclusion:** These findings support the hypothesis that in FM a deteriorated function of cortical inhibition, indexed by a higher SICI parameter, a lower function of the DPMS, together with a higher level of BDNF indicate that FM has different pathological substrates from depression. They suggest that an up-regulation phenomenon of intracortical inhibitory networks associated with a disruption of the DPMS function occurs in FM.

## Introduction

Major depressive disorder (MDD) and fibromyalgia (FM) present overlapped symptoms. Although the connection between these two disorders has not been elucidated yet, the disruption of neuroplastic processes that mediate the equilibrium in the inhibitory systems stands out as a possible mechanism. These processes comprise a central pathologic mechanism of the sensitization syndrome (CSS) (Maletic et al., 2007; Woolf, 2012). The CSS embodies the long-term consequence of an abnormal stress-response system (Lyon et al., 2011) that culminates in the amplification of sensory inputs. It covers the decline of top-down inhibitory activity (dysregulation of dopamine, serotonin, norepinephrine, epinephrine, and endogenous opioids) (Wallace and Gotto, 2008) and the enhancement of bottom-up excitatory activity.

Both MDD and FM present a robust association with an imbalance of glutamatergic (Glu) and GABAergic transmission. Motor cortex disinhibition indexed by transcranial magnetic stimulation (TMS) measurements became a robust common feature of MDD (Fidalgo et al., 2014; Lewis et al., 2016) and FM (Caumo et al., 2016). In chronic pain, changes in the short intracortical inhibition (SICI) (a surrogate marker of GABAergic activity) are mixed. Some studies in neuropathic pain (Nijs et al., 2014), chronic myofascial, FM, and migraine found a reduction in the SICI (Chadaide et al., 2007; Dall’Agnol et al., 2014). And, a similar result has been found in depression (Antal et al., 2010; Conforto et al., 2012; Cantone et al., 2017). Regarding to intracortical facilitation (ICF) (a proxy of glutamatergic activity), an increased activity of excitatory intracortical interneurons (Dall’Agnol et al., 2014; Vidor et al., 2014; Botelho et al., 2016; Caumo et al., 2016; Dussán-Sarria et al., 2018) was found in chronic pain, while it is decreased in MDD (Cantone et al., 2017). Another biomarker associated with both MDD and FM is the brain-derived neurotrophic factor (BDNF) (Zhou et al., 2017). A reduction of the serum BDNF has been observed in MDD (Zhou et al., 2017), while an increment has been found in FM (Zanette et al., 2014; Deitos et al., 2015; Caumo et al., 2016).

The BDNF has a central role in the clinical picture of dysfunctional neuronal circuits. It strengthens glutamatergic synapses, while it weakens GABAergic synapses (Zhang et al., 2013). In chronic musculoskeletal pain, the serum BDNF was inversely correlated with the SICI and positively correlated with a decreased inhibitory role of the descending pain modulatory system (DPMS). Thereby, it is reasonable to consider the serum BDNF and the motor cortex excitability measured by TMS as probing neural plasticity indexes to improve the comprehension of the neural substrates shared by FM and MDD, as well as their interplay with the inhibitory function of DPMS. The DPMS function is assessed by the conditioned pain modulation (CPM) paradigm. CPM engages activation of a cortically regulated spinal-bulb-spinal loop by the diffuse noxious inhibitory control (DNIC) mechanism, where “pain-inhibits pain” phenomenon (Bars et al., 1979; Yarnitsky, 2010). The neurobiological mechanism involved in the CPM-test includes several neurobiological systems, such as serotoninergic, opioidergic, and noradrenergic (Lindstedt et al., 2011; Baba et al., 2012; Treister et al., 2013). These neurobiological systems are also involved with psychological characteristics of chronic pain, i.e., anxiety (Karg et al., 2011; Horjales-Araujo et al., 2013), depression (Karg et al., 2011), and pain catastrophizing (Horjales-Araujo et al., 2013). Thus, the DPMS is also influenced by psychological characteristics, which explain at least part of the interpersonal variability in pain perception (Racine et al., 2012). According to earlier studies, a higher score on the CS Inventory for chronic pain was positively associated with greater dysfunction of DPMS and correlated positively with serum BDNF (Caumo et al., 2017). While another study with in chronic myofascial pain found a positive association of DPMS with increase in ICF, serum BDNF levels, and disability due to pain (Botelho et al., 2016). At the same way in chronic pain (e.g., FM and chronic myofascial pain) compared to osteoarthritis the SICI was associated with greater dysfunction in DPMS (Caumo et al., 2016). We hypothesize that a deteriorated function of cortical inhibition, the dysfunction of the inhibitory DPMS and serum BDNF can differentiate FM from MDD and HS.

Considering that homeostatic plasticity is the ability of neurons to maintain their levels of excitability within a narrow range, thereby, the disruption of this equilibrium is likely to have a central role in the physiopathology of FM and MDD. Thus, this exploratory study tested the hypothesis that FM patients would present higher disinhibition of the motor cortex compared to MDD and HS. Another hypothesis was that the dysfunction of the DPMS is related to the disinhibition of the motor cortex evaluated by the SICI in FM. Thus, this study was meant to meet the following objectives: (i) to compare the motor cortex inhibition indexed by the TMS measures SICI and ICF as well as the DPMS to evaluate the neuroplastic changes in FM, MDD, and HS; (ii) to compare the inhibitory function at the cortical level indexed by the SICI and ICF as well as the descending pain inhibitory system between clinical diagnoses (FM and MDD) considering the BDNF adjusted index as a marker of neuroplasticity; (iii) to examine the relationship between the role of DPMS with the BDNF adjusted index despite clinical diagnosis.

## Materials and Methods

### Study Design, Settings, and Subjects

We conducted an exploratory cross-sectional study following the Strengthening the Reporting of Observational studies in Epidemiology (STROBE) statement. The Ethics Committee Board of the Hospital de Clínicas de Porto Alegre (HCPA) (Institutional Review Board IRB 0000921) approved the protocol. All individuals provided oral and written consent before their engagement in the study.

### Participants

**FIGURE 1:**
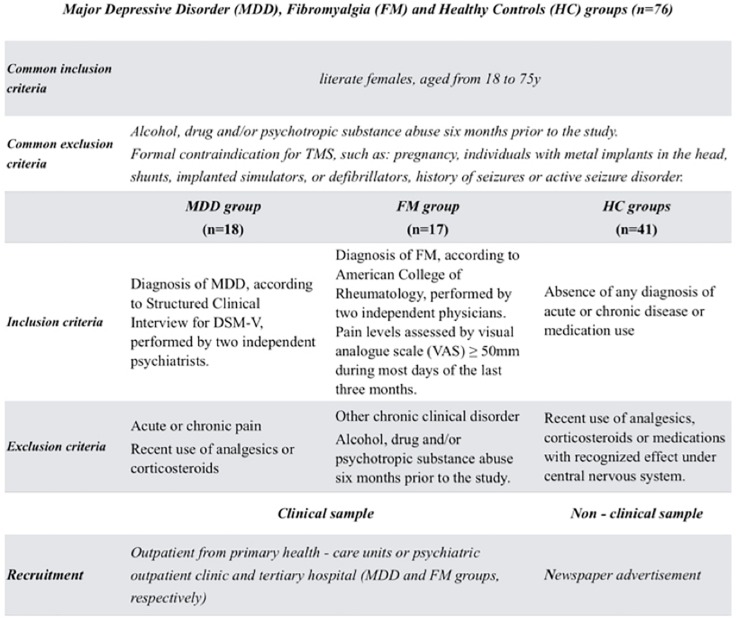
The flow of the study with the inclusion and exclusion criteria of three groups: patients with depression or fibromyalgia and healthy subjects.

The study’s subject recruitment and data collection were conducted from August 2017 to July 2018. The sample included literate, right-handed females, aged from 18 to 75 years. The inclusion and exclusion criteria pertaining each one of the three groups (FM, MDD, and HC) are presented in Figure 1.

Major depressive disorder subjects were recruited from the Basic Health Unit (BHU). Diagnosis of MDD was performed according to the diagnostic criteria outlined in the Diagnostic and Statistical Manual of Mental Disorders, 5th Edition (American Psychiatric Association, 2013).

Fibromyalgia patients were recruited by direct contact from the institutional chronic pain clinic, by referrals from other clinic units, and from the BHU at HCPA. They were reached by phone and answered a screening questionnaire. Those who met the inclusion criteria were invited to medical evaluation for history collection and a detailed description of their symptoms. FM diagnosis was established according to the criteria of the American College of Rheumatology (2010–2016) by experienced pain physicians. Those patients who reported at least a pain score on the Numerical Pain Scale (NPS) greater than 5, on most days of the last 3 months, were included.

Volunteers were recruited from the community by advertisement postings in universities and in public places in Porto Alegre city. They answered a structured questionnaire that assessed the following variables: current acute or chronic pain conditions, use of analgesics in the past week, rheumatologic disease, clinically significant or unstable medical psychiatric disorder, history of alcohol abuse in the past 6 months, neuropsychiatric comorbidity, and use of psychotropic substance or drugs. They were excluded when presenting scores higher than 13 on the Beck Depression Inventory (BDI) (Beck et al., 1996; Gomes-Oliveira et al., 2012).

### Instruments and Assessment

#### Outcomes

The primary outcome was the motor cortex inhibitory function indexed by SICI and ICF, and the DPMS assessed by the change on NPS during CPM-test, ranging from 0 to 10 during CPM-test. A secondary outcome was the heat pain threshold (HPT).

#### Dependent Variables

##### TMS measures

Left primary motor cortex (M1) measures were assessed through TMS MagProX100 stimulator (MagVenture Company, Lucernemarken, Denmark) with figure-eight coil (MagVenture Company). Ag-AgCl electrodes were placed over the first dorsal interosseous (FDI) belly muscle and in its corresponding tendon on the distal phalanx of the index finger. Then, we recorded the responses to stimuli from the FDI muscle of the right hand by surface electromyography (EMG). Before to start the measures, patients were placed in a comfortable chair and informed about the TMS procedure, including possible sensations that might experience.

To identify motor “hot spot,” the coil was placed over the left M1 at 45°angle to the sagittal line tangential to the scalp. To ensure the proper placement of the coil during cortical excitability assessments, researchers marked the site with a soft-tipped pen. To reduce variability, the same researcher performed all TMS assessments. The measures of TMS, such as amplitudes of the single and paired-pulse, and the latency and the measures of the cortical silent period (CSP) were recorded on an Excel spreadsheet.

Motor threshold (MT) defined as the lowest stimulus used to induce 50% of the evoked potentials of resting FDI. Initially, it was set the minimum amplitude of 50 mV peak-to-peak, in at least 5 of 10 (at least 50%) of successive trials. Subsequently, single-pulse TMS protocol with an intensity of 130% of MT was applied to record ten motor evoked potential (MEP). It is a measure that reflects the excitability of the membrane potential of pyramidal neurons in M1 (Nielsen and Norgaard, 2002).

Cortical silent period has been associated with inhibitory network influenced by GABAB-receptors (Werhahn et al., 1999). Likewise, ten CSPs (measured during muscle activity measured on a dynamometer set to approximately 20% of the maximal force) were recorded using an intensity of 130% of the RMT.

Short intracortical inhibition mainly reflects GABA(A) receptor-mediated inhibitory function (Ilić et al., 2002; Cash et al., 2017), while ICF denotes excitatory transmission mostly through the glutamatergic N-methyl-D-aspartate receptor (Ziemann et al., 1998).

We used a paired-pulse TMS protocol to measure SICI and ICF with an interstimulus interval (ISI) to evaluate the SICI equal to 2 and 12 ms for ICF, respectively.

We set individual conditioning stimulus (first) at 80% of the MT to measure the ICF and SICI, while for the test stimulus (second) we set at 130% (Kujirai et al., 1993a). A total of 30 randomized paired-pulse trials were conducted (ten for each measure: SICI, ICF, and control stimuli). The units for these parameters were: MEP in mV; SICI and ICF in their ratio to the MEP; and the CSP in milliseconds (ms) (Pascual-leone et al., 1994).

##### Conditioned pain modulation test (CPM-test)

Conditioned pain modulation test (CPM-test) to evaluate the DPMS a nociceptive tonic stimulus was used, such as immersion of the non-dominant hand in water at a temperature of zero up to 1°C for 1 min. To control if water temperature was maintained in the range between zero to 1°C, a thermostat was used to control temperature variation. The QST procedure was introduced after 30 s of cold-water immersion. The CPM TEST was determined by the difference between the pain score on NPS during the QST at the same time they maintained their dominant hand in cold water immersion (QST+CPM) than the temperature of the subjects felt 6/10 pain on the NPS scale [during the initial period (T0)].

##### Heat pain threshold

It was assessed through quantitative sensory testing (QST), which uses the method of limits with a computer Peltier-based device thermode (30630 mm) (Schestatsky et al., 2011). Firstly, the thermode was attached to the skin on the ventral position of the mid-forearm. Baseline temperature was set at 32°C and increased at a rate of 1°C/s to a maximum of 52°C. Each participant was instructed to push the button immediately at the moment the stimulation became painful. This trial was composed of three assessments with an ISI of 40 s (Schestatsky et al., 2011), and then an average temperature of the three assessments was calculated. The position of the thermode was slightly altered between evaluations to avoid sensitization of nociceptors.

### Independent Variables

#### Assessments of Demographic and Clinical Characteristics

##### Standardized questionnaire

A standardized query was used to assess demographic data and medical comorbidities. Patients were requested to provide information about their age, sex, level of education, marital status, and lifestyle habits. They also provided information about their health status, including clinical and psychiatric diagnosis.

##### Psychiatric diagnosis

Psychiatric diagnosis was based on the Structured Clinical Interview for DSM-V (SCID) applied by a trained psychiatrist. This instrument consists of a semi-structured diagnostic interview created from DSM – V. The answers identify the presence or absence of the symptoms, scored according to the judgment of the evaluator. It is composed of 10 modules, which can be used in a combined or independent way (2012). In the study, the “A” module was used to diagnose mood episodes (MDDs). The translation and adaptation of this clinical interview into Portuguese language present, in general, good reliability for mood disorders (Del-Ben et al., 2001).

##### Psychological state and sleep quality

All instruments used were validated for the Brazilian population and the assessments were conducted by two trained evaluators. The following tools were applied: Beck Depression Inventory-II (Beck et al., 1996; Gomes-Oliveira et al., 2012), Pittsburgh Sleep Quality Index (PSQI) (Buysse et al., 1989; Bertolazi et al., 2011), Fibromyalgia Impact Questionnaire (FIQ) (Burckhardt et al., 1991; Marques et al., 2006), State-Trait Anxiety Inventory (STAI) (Kaipper et al., 2010), Brazilian Portuguese Pain-Catastrophizing scale (BP- PCS) (Sehn et al., 2012) and Visual Analog Pain Scale (0 no pain and ten worst pain). Analgesic use was defined by an average of analgesics used per week during the previous month. For data analysis, analgesic use was included as a dichotomous variable (more than 4 days per week or lower than 4 days per week).

##### BDNF dosage

Blood samples were collected and identified in a standardized manner. The blood samples were obtained in plastic tubes and centrifuged for 10 min at 4,500 rpm at 4°C. Serum was stored at −80°C for further BDNF assay. Serum-mediator concentrations were determined using BDNF (Chemicon CYT306, lower detection limit 7.8 pg/mL; EMD Millipore, Billerica, MA, United States) enzyme-linked immunosorbent assay kits, according to the manufacturer’s instructions.

#### Efforts to Address Potential Sources of Bias

In order to reduce assessment bias, two researchers with the vast clinical expertise in treating outpatients in pain clinic were responsible for making the diagnostics according to pre-specified criteria. A trained psychiatrist with more than 10 years of experience in psychiatric care applied the psychiatric diagnosis based on the SCID-VR. Two evaluators with specific training were responsible for all assessments and for applying the standardized protocol to assess the QST and the CPM-test. To reduce the variability, the same researcher performed all TMS assessments.

### Study Size

For type I and II errors of 0.05 and 0.20, respectively, and anticipating partial η^2^ of 0.25 for multiple regression analysis, which allows for three predictors (diagnosis, age, and BDNF), a sample size of 60 patients was estimated. It was calculated using the *post hoc statistical power calculator for hierarchical multiple regression* at https://www.danielsoper.com/statcalc/calculator.aspx?id=17. Finally, considering the likely attrition rate and other unexpected factors, the required sample size was determined to be 63 patients.

### Statistical Analysis

To assess if the data presented a normal distribution the Shapiro was used – Wilk test. Descriptive statistics were used to summarize the main characteristics of the sample. ANOVA was performed to compare the three groups in the univariate analysis. A MANCOVA was used to test the differences between groups (FM, MDD, and healthy controls) on the multiple outcome controlled for age (Huberty and Morris, 1989). The dependent variables included in the MANCOVA were the cortical excitability [SICI and ICF and the change on the NPS (0–10) during the CPM-test] and HPT (secondary outcomes).

To construct an adjusted surrogate index of factors related to neuroplasticity we created a BDNF adjusted index (dependent variable). For this purpose, we used a multivariate regression model with a stepwise method controlled by multicollinearity. We included in the model the following variables, which can affect the biological process of BDNF secretion: age, analgesic use, classes of antidepressants: [selective serotonin reuptake inhibitors (SSRIs), serotonin–norepinephrine reuptake inhibitors (SNRIs), tricyclic] and anticonvulsants uses (Yes/No).

Another MANCOVA model was used to assess the relationship between the SICI, ICF and the change on NPS during CPM-test (dependent variables) with the BDNF – adjusted index as a covariate, according to diagnosis group. To analyze the correlation between the SICI, change on NPS during CPM-test and BDNF adjusted index the Spearman’s correlation analysis was used. All analyses were adjusted by multiple comparisons using the Bonferroni’s Multiple Comparison Test. To analyze the data, we used the software SPSS version 22.0 (SPSS, Chicago, IL, United States).

## Results

### Socio-Demographic, Clinical, and Psychological Characteristics of the Sample

The demographic, the clinical and the psychiatric characteristics are presented in Table 1. The analysis showed that compared to controls, both MDD and FM groups are older and have lower educational levels. In comparison to healthy controls, both FM and MDD presented higher levels of trait anxiety and depressive symptoms.

**TABLE 1:** Demographic characteristics.

	Fibromyalgia	Major depressive disorder	Healthy control
	(*n* = 17)	(*n* = 18)	(*n* = 28)
***Demographic***			
Age (years)*	50.5 (±8.7)^a^	45.2 (±15.9)^a^	43.8 (±13.0)^b^
BMI^2^* (Kg/m^2^)	31.31 (±7.3)^a^	25.89 (±5.2)^b^	22.78 (±2.9)^b^
Years of education [median (Q25–75)]°	11.0 (6.5–12.5)^a^	11.5 (10.0–16.2)^a^	17.0 (15.7–18.5)^b^
Employed (yes/no)	10/7	14/4	41/0
Smoking (yes/no)	4/13	1/17	1/40
Alcohol use (yes/no)	4/13	7/11	12/29
***Clinical and psychiatric***			
Use of psychotropic medications (yes/no)	11/6	18/0	
Selective serotonin reuptake inhibitors (SSRIs) (yes/no)	11/6	16/02	
Tricyclic antidepressant (yes/no)	10/7	10/8	
Dual antidepressant (yes/no)	2/15	2/16	
Pregabalin (yes/no)	6/11	0/18	
Antipsychotic (yes/no)	0/17	3/15	
Clinical chronic disease (yes/no)*	11/6^a^	11/7^a^	1/40^b^
*Hypertension (yes/no)*	10/7	3/15	1/40
*Type 2 Diabetes mellitus (yes/no)*	1/16	3/15	
*Asthma (yes/no)*	3/14	1/17	
Psychiatric disorder according to the SCID-V (yes/no)*	12/5	18/0	
*Major depressive episode*	12/5	18/0	
*Generalized anxiety disorder*	3/14	1/17	
Beck Depression Inventory – BDI – II*	25.4 (±12.8)^a^	22.3 (±14.4)^a^	3.4 (±4.5)^b^
Pain Catastrophizing Scale – PCS*	33.9 (±12.0)^a^	15.7 (±13.6)^b^	6.1 (±8.0)^c^
State-Trait Anxiety Inventory – STAI*			
STAI – State*	27.3 (±5.3)^a^	28.4 (±3.6)^a^	28.4 (±3.6)^a^
STAI – Trait*	29.35 (±8.1)^a^	27.4 (±4.5)^a^	22.1 (±5.3)^b^
Pittsburgh Sleep Quality Index – PSQI*	12.6 (±4.8)^a^	7.1 (±2.3)^b^	3.74 (±2.0)^c^
Fibromyalgia impact questionnaire (FIQ)	70.4 (±14.5)	–	–
***Pain measures***			
Analgesic doses used per week median (Q25–75)°	16 (6.5 – 24.5)	–	–
Pain on the VAS (0 – 100 mm) median (Q25–75)°	6.7 (5.8 – 8.2)	–	–
Quantitative sensory testing (QST)			
QST: heat pain threshold*	38.0 (±3.5)^a^	39.8 (±3.7)^a,b^	42.1 (±3.2)^b^
Pressure pain threshold (kg/cm^2^)*	2.4 (±1.1)^a^	4.1 (±1.3)^a^	4.1 (±1.3)^b^

Data area presented as mean and standard deviation (SD) or frequency (*n* = 63). ^2^Body Mass Index. *Comparisons using ANOVA. *Post hoc* differences from each other are indicated via superscript numbers. °Comparison by Kruskal–Wallis Test, values represented as median and P25 – P75 comparisons using ANOVA. *Post hoc* differences from each other are indicated via superscript numbers.

The cortical excitability parameters measured by TMS, psychophysical measures and serum BDNF according to diagnosis group are presented in Table 2. We observed that FM group, compared to MDD, showed lower ICF, higher SICI, and higher serum BDNF. However, in this univariate analysis, we did not find a difference in the efficiency of DPMS among groups.

**TABLE 2:** Cortical excitability measures assessed by the TMS, psychophysical pain measures, and BDNF.

	Fibromyalgia (*n* = 17)	Major depressive disorder (*n* = 18)	Healthy control (*n* = 28)	*F*	*P*
Motor evocate potential – MEP	1.28 (±0.25)	1.56 (±0.52)	1.45 (±0.43)	1.750	0.183
Intracortical facilitation – ICF	0.33 (±0.23)^a^	1.39 (±1.02)^b^	1.14 (±0.27)^b^	16.268	<0.001
Short intracortical inhibition – SICI	1.03 (±0.50)^a^	0.55 (±0.43)^b^	0.34 (±0.19)^b^	18.701	<0.001
Cortical silent period – CSP	67.21 (±19.51)^a^	48.58 (±12.21)^b^	70.90 (±25.38)^a^	8.168	0.001
BDNF ng/ml	49.82 (±16.31)^a^	14.12 (±8.86)^b^	18.04 (±10.19)^b^	50.246	<0.001
Heat pain threshold (C)	38.03 (±3.45)^a^	39.83 (±3.70)^a,b^	42.11 (±3.23)^b^	7.903	0.001
Change on NPS during CPM TEST	0.22 (±1.37)^a^	−0.87 (±1.49)^a^	−2.54 (±2.46)^b^	11.208	<0.001

*Data are presented as mean and standard deviation (SD) (*n* = 63). Different superscripts (a,b) indicate significant difference among treatment groups after *post hoc* analysis adjusted by Bonferroni (*P* < 0.05). Analysis of variance (ANOVA) to compare mean (SD).*

### Assessment of Cortical Excitability (SICI, ICF), and HPT According to Groups

A MANCOVA with the cortical excitability (SICI and ICF), the function of DPMS assessed by the change on NPS during CPM-test and HPT parameters as dependent variables and independent age revealed a significant difference between groups (Hotelling’s Trace = 0.99, *F* = 10.42, and *P* < 0.0001). FM group compared to healthy controls showed lower HPT, higher SICI, and lower ICF. While the MDD group compared to healthy controls presented larger SICI. However, MDD did not show a difference in the ICF. The age did not correlate with the SICI, ICF, and HPT. The results of this adjusted multivariate model are presented in Table 3.

**TABLE 3:** Multivariate linear regression model of the cortical excitability and heat pain threshold measures among FM, MDD, and HC groups (*n* = 63).

Dependent variables		Type III sum of squares		*df*	Mean square	*F*	*P*	Partial eta squared
**(A) Main effects**							
**Corrected model**							
Heat pain threshold (°C degree)		33.462^a^		3	11.154	7.269	0.000	0.326
Change on NPS during CPM-test		53.593^b^		3	17.864	5.129	0.004	0.255
Short intracortical inhibition [(SICI), ratio: SICI/test stimulus]		4.255^c^		3	1.418	7.923	0.000	0.346
Intracortical facilitation [(ICF), ratio: ICF/ test stimulus]		11.506^d^		3	3.835	8.972	0.000	0.374

	***B***	***SEM***	***t***		***P***		***CI 95%***	

**(B) Beta coefficients**					
**Dependent variable: heat pain threshold (°C degree)**					
*Intercept*	3.387	0.699	4.844		0.000		(1.97 to 4.79)	
Fibromyalgia	−1.816	0.457	−3.973		0.000		(−2.74 to −0.89)	
Major depressive disorder	−0.067	0.442	−0.153		0.879		(−0.95 to 0.82)	
Healthy controls	0^reference^							
Age	0.017	0.014	1.232		0.224		(−0.01 to 0.05)	
**Dependent variable: Change on NPS during CPM-test**								
*Intercept*	−3.104	1.053	−2.947		0.005		(−5.22 to −0.98)	
Fibromyalgia	2.394	0.689	3.476		0.001		(1.07 to 3.78)	
Major depressive disorder	1.335	0.666	2.005		0.051		(−0.06 to 2.67)	
Healthy controls	0^reference^							
Age	0.022	0.021	1.055		0.297		(−0.02 to 0.07)	
**Short intracortical inhibition [(SICI), ratio: SICI/test stimulus]**								
*Intercept*	0.245	0.239	1.026		0.310		(−0.24 to 0.73)	
Fibromyalgia	0.698	0.156	4.469		0.000		(0.38 to 1.02)	
Major depressive disorder	0.215	0.151	1.428		0.160		(−0.09 to 0.52)	
Healthy controls	0^reference^							
Age	0.002	0.005	0.428		0.671		(−0.008 to 0.01)	
**Dependent variable: intracortical facilitation [(ICF), ratio: ICF/ test stimulus]**								
*Intercept*	1.618	0.369	4.385		0.000		(0.87 to 2.36)	
Fibromyalgia	−0.731	0.241	−3.030		0.004		(−1.22 to −0.25)	
Major depressive disorder	0.273	0.233	1.169		0.249		(−0.19 to 0.74)	
Healthy controls	0^reference^							
Age	−0.011	0.007	−1.496		0.142		(−0.02 to 0.004)	

*^a^*R*^2^ = 0.326 (Adjusted R^2^ = 0.282). ^b^*R*^2^ = 0.255 (Adjusted *R*^2^ = 0.205). ^c^*R*^2^ = 0.346 (Adjusted *R*^2^ = 0.302). ^d^*R*^2^ = 0.374 (Adjusted *R*^2^ = 0.333).*

### Relationship Between Cortical Excitability and Descendent Pain Modulatory System With the BDNF According to MDD and Fibromyalgia

Factors such as age, antidepressant, anticonvulsant, antipsychotic and analgesics can influence either BDNF secretion, the cortical excitability or the function of descending pain modulating system. They are involved in the neuroplasticity processes. Thus, we construct a BDNF adjusted index as a surrogate marker of the neuroplasticity. For this purpose, the multiple regression analysis was used. The variables antidepressant selective serotonin reuptake inhibitors (SSRIs), anticonvulsants and analgesic use were retained in the model. Age and tricyclic antidepressant were excluded from the model. The mean (SD) of serum BDNF according to SSRIs users and no-users was 27.77 (5.63) vs. 43.91 (25.63), respectively. The *R*^2^ = 0.38, [β coefficient = −14.50, confidence interval (CI) 95% = −26.43 to −2.56, *t* = 2.48]. Whereas, the mean (SD) of serum BDNF according to anticonvulsant use or not was 60.87 (15.54) vs. 25.37 (18.18), respectively. The *R*^2^ = 0.54, (β coefficient = 22.71, CI 95% = 8.19 to 37.22). The mean (SD) when they used analgesics more than 4 days per week or lower than 4 days per week was 48.03 (17.51) vs. 20.41 (17.79), respectively. The *R*^2^ = 0.54, (β coefficient = 20.94, CI 95% = 9.84 to 32.04).

A MANCOVA model was used to assess the relationship of dependent variables (SICI, ICF, and CPM-test) according to FM and MDD groups adjusted by the BDNF adjusted index. This analysis revealed a significant difference between diagnostic groups (Hotelling’s Trace = 0.70, *F* = 7.06, and *P* = 0.001). The BDNF adjusted index did not correlate with the SICI, ICF, nor with the change on NPS during CPM-test. The power of this analysis was 96%. The results of this adjusted multivariate model are presented in Table 4. The analysis revealed that the FM group compared to MDD showed a greater dysfunction of the descending pain inhibitory system compared to MDD. However, FM showed higher SICI compared to MDD, in the sense that there is a disengagement between the inhibitory motor cortex function and the descending pain inhibitory system. Whereas, we did not find a difference between groups in the ICF.

**TABLE 4:** Relationship between intracortical inhibition (SICI and ICF) and descendent pain modulating as assessed by the change on NPS during CPM-test with the BDNF according to diagnosis group (FM and MDD) (*n* = 35).

	Type III sum of squares	*df*	*F*	Mean square error		*P*		Partial eta squared
***Corrected model***								
Intracortical facilitation (ICF)	10.755^a^	2	5.377	9.751		0.000		0.38
Change on NPS during CPM TEST	13.812^c^	2	6.906	3.907		0.030		0.20
Short intracortical inhibition (SICI)	2.110^b^	2	1.055	4.786		0.015		0.23

	***B***		**SEM**	***t***	***P***		***CI 95%***	

**Dependent variable: intracortical facilitation [(ICF), ratio: ICF/ test stimulus]**								
*Intercepted*	1.662		0.278	5.978	0.000		(1.09 to 2.23)	
Fibromyalgia	−0.641		0.419	−1.531	0.136		(−1.49 to 0.21)	
Major depressive disorder	0^reference^							
*BDNF – adjusted – index*	−0.015		0.012	−1.260	0.217		(−0.04 to 0.009)	
**Dependent variable: change on NPS during CPM-test**								
*Intercepted*	−0.385		0.498	−0.773	0.445		(−1.39 to 0.63)	
Fibromyalgia	1.760		0.749	2.349	0.025		(0.23 to 3.28)	
Major depressive disorder	0^reference^		.	.	.		.	
*BDNF – adjusted – index*	−0.021		0.022	−0.970	0.339		(−0.07 to 0.02)	
**Short intracortical inhibition [(SICI), ratio: SICI/test stimulus]**								
*Intercepted*	0.659		0.176	3.752	0.001		(0.30 to 1.02)	
Fibromyalgia	0.640		0.265	2.418	0.021		(0.10 to 1.18)	
Major depressive disorder	0^reference^		.	.	.		.	
*BDNF – adjusted – index*	−0.006		0.008	−0.77	0.443		(−0.021 to 0.01)	

*^a^*R*^2^ = 0.379 (Adjusted *R*^2^ = 0.340). ^b^*R*^2^ = 0.230 (Adjusted *R*^2^ = 0.182). ^c^*R*^2^ = 0.196 (Adjusted *R*^2^ = 0.146).*

**FIGURE 2:**
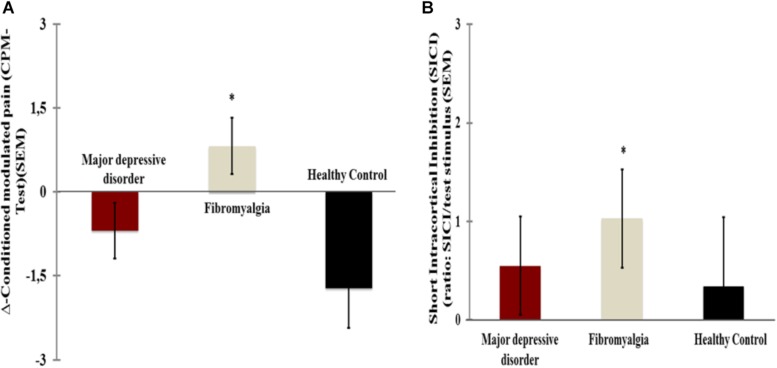
Comparisons between [fibromyalgia (*n* = 17) and major depressive disorder (*n* = 18)]. **(A)** Short intracortical inhibition [(SICI) ratio: SICI/test stimulus]. **(B)** Change on Numerical Pain Scale (NPS) during CPM TEST. Error bars indicate standard error of the mean (S.E.M.). *Positioned above the bars indicate differences between groups (fibromyalgia and major depressive disorder) assessed by MANCOVA with *post hoc* Bonferroni’s multiple comparison test.

Figures 2A,B present the relationships between the SICI and the CPM (primary outcomes) according to FM and MDD. The means were compared using MANCOVA, and *post hoc* adjusted for multiple comparisons using Bonferroni correction (the model is presented in Table 4).

### Secondary Analysis: Relationship Between SICI, Change on NPS During CPM-Test and BDNF Adjusted Index

**FIGURE 3:**
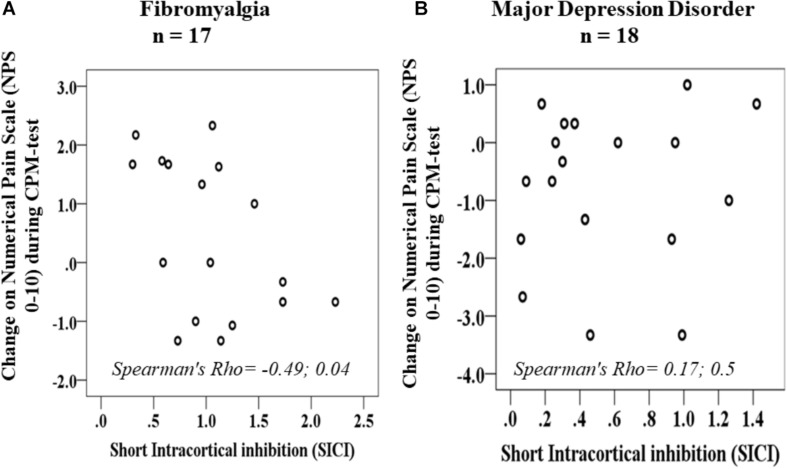
Scatter plots of change on NPS during CPM-test and SICI according to fibromyalgia **(A)** and major depressive disorders **(B)**.

The Scatter plots of the raw change on NPS during CPM-test and SICI according to diagnosis group FM and MDD is presented in Figures 3A,B, respectively. The change on NPS during CPM-test and SICI in the FM showed a conversely non-parametric correlation. Such non-parametric correlation means that in patients with FM a greater SICI is related to lower scores in the CPM-test or vice – versa. It is important to highlight that lower scores in the CPM-test indicates better function of the DPMS as assessed by the change on the NPS during CPM-test. The correlation coefficient between the scores in the NPS (0 – 10), during CPM TEST and the SICI in the FM was Spearman’s Rho = −0.49 and its CI 95% was (−0.78 to −0.03); *P* = 0.04. The correlation coefficient between the NPS, during CPM-test and the SICI in the MDD was Spearman’s Rho = 0.17, and its CI 95% was (−0.32 to 0.59); *P* = 0.5.

The BDNF adjusted index and change on NPS during CPM-test, despite diagnosis group, showed a positive non-parametric correlation. Such non-parametric correlation means that a greater score in the BDNF adjusted index was correlated with higher dysfunction of the DPMS or vice-versa. The Spearman’s Rho = 0.35, and its CI 95% was (0.02 to 0.61); *P* = 0.03.

## Discussion

These results extent evidence that FM displays a deteriorated function of cortical inhibition, indexed by higher SICI parameter compared to MDD and HC. This finding contrasts to our initial hypothesis that it would be decreased. On the other hand, it confirms the assumption that there is greater disinhibition of the DPMS in FM compared to MDD and that it is conversely correlated with the SICI in FM but not in MDD. Also, they showed a positive relationship between the change in the NPS during CPM-test with a measure of neuroplasticity composed by the BDNF adjusted index, despite the clinical diagnostic.

These results demonstrate the relevance of using the motor cortex measures to understand the imbalanced inhibitory or excitatory intracortical neurochemical circuitry to comprehend the underpinning pathophysiology process of FM and MDD. The most relevant result was to show that the increase of SICI is conversely correlated with the change in the NPS during CPM-test only in FM, in the sense that more substantial intracortical inhibition is associated to a higher potency of the descending pain inhibitory system. Here, it is important to realize that negative values in the CPM-test indicate a higher effect of heterotopic stimulus inhibits the test stimulus (i.e., “pain-inhibits pain”), in other words, a better function of the DPMS. Indeed, the change in the SICI may indicate that a disruption of mechanism mediated by inhibitory gamma-aminobutyric acid (GABAergic) occurs interneurons within the primary motor cortex (Di Lazzaro et al., 2006) in FM, at the same time, it indicates an up-regulation phenomenon of intracortical inhibitory networks mediated by GABAA receptors. As previously demonstrated by the tiagabine use (a GABA-reuptake inhibitor) that decreased the SICI (Werhahn et al., 1999) or reduced excitability of intracortical facilitatory systems (van Elswijk et al., 2007). In the same way, earlier studies found that changes in pain pathways that facilitate convergent stimuli are associated with nerve injury, which can induce selective apoptosis of inhibitory GABAergic interneurons (Moore et al., 2002). These processes decreased the inhibitory receptors expression on primary afferent terminals and postsynaptic neurons, and it culminates with a higher perception of repetitive nociceptive stimuli (Staud et al., 2007). Both phenomena comprise a primary mechanism of the pathophysiology of neuropathic pain syndromes, but it also has been pointed out as a possible mechanism in FM, while the CPM-test is a marker of dysfunction of DPMS in chronic pain. Thus, this increased cortical inhibition could be a compensatory response to contra-regulate the changes induced by the persistent hyperexcitability induced by chronic pain. This hypothesis finds support in a physiological protective reaction, when a prolonged effort at adaptation can result in the dysregulation of other systems, such as autonomic, metabolic, and inflammatory systems. Also, this assumption is substantiated by results of the previous study in chronic pain syndromes (trigeminal neuralgia, poststroke pain syndrome, back pain, and FM), which demonstrated a decreased intracortical inhibition after anodal stimulation, concurrently with the improvement in pain scores (Antal et al., 2010). Likewise, the rTMS induced a long-lasting reduction in the SICI by a possible mechanism mediated by activation of NMDA receptors associated to downregulate hyper excitability associated with the mal-adaptive neuroplasticity (Kobayashi and Pascual-Leone, 2003).

In contrast, observational studies found a decreased SICI in chronic pain (Mhalla et al., 2010). Although the reasons to explain these differences are not clear, it is necessary to consider that the FM is a syndrome with complex pathophysiology involving a neurochemical imbalance in the excitatory and inhibitory mechanisms mediated by multiple systems (i.e., GABAergic, glutamatergic, noradrenergic, serotonergic, etc.). Likewise, it is possible that these incongruences between the results of studies may be explained by the sample characteristics related to the severity of disorders, the medication used, disability, comorbidities, etc.

This difference in the SICI between FM and depression suggest that these two disorders may have considerable overlaps in neuroplasticity processes, but the TMS patterns together with the distinct standard of dysfunction in the DPMS as well in the BDNF serum indicate that these two disorders have substantial differences in their pathophysiological mechanisms. This way, these results give support to understand differences in the cardinal symptoms of each one of these two disorders (i.e., fatigue, migratory pain, pain catastrophizing, etc.), which are prototypical symptoms of FM. Besides, it can help to personalize the therapeutic approach. Despite the absence of a FDA-approved neuromodulation protocol targeted to patients with comorbidity MDD and FM, the effectiveness of neuromodulatory techniques (i.e., tDCS and TMS) has been supported by consistent evidence to treat both FM and depression (Kauffmann et al., 2004; Antal et al., 2010; Brunoni et al., 2011; Marie, 2014; Fagerlund et al., 2015; Castillo-Saavedra et al., 2016; Cheng et al., 2018; Karina do Monte-Silva et al., 2019). Considering that these two disorders are frequently overlapped, it poses a considerable challenge to decide if it would be better to stimulate the M1 or the dorsal lateral prefrontal cortex for the treatment of pain accompanied by depression.

However, the interpretation of the SICI measure should consider that it is a low-threshold inhibition test elicited during paired-pulse TMS, which does not influence the descending corticospinal volleys (Di Lazzaro et al., 1999) neither alters spinal reflexes (Kujirai et al., 1993b). Thus, the SICI might be a tool to identify the cortical inhibition. However, the values of SICI should not be interpreted in isolation, since it is influenced by several factors such as the precision of measurement, the mechanism of pain (i.e., inflammatory vs. neuropathic pain), the severity of pain and the psychotropic medications, etc., Accordingly, the M1 may be an entry port to assess the complex pain-related neural network, also to understand the M1 role to inhibit or interrupt pain signals and as a measure to evaluate the cortical process on the neuroplasticity of chronic pain. This hypothesis finds support in a previous study, which showed that in FM a strong M1–ventral lateral thalamus connectivity at baseline predicted a more significant reduction in pain across tDCS treatment (Cummiford et al., 2016). A similar effect was found when the invasive chronic motor cortex stimulation decreased the thalamic hyperactivity in patients with thalamic pain (Tsubokawa et al., 1993). Aligned with this assumption, we found extensive literature showing that the transcranial stimulation (i.e., tDCS and TMS) might improve the disrupted neurochemical processes in chronic pain (Cheng et al., 2010).

Both FM and MDD are disorders associated with chronic stress that share several symptoms and sometimes co-exist in the same patient. In this study greater serum levels of BDNF in FM compared to MDD and healthy controls was observed. The current finding is in agreement with the previous studies that found higher serum BDNF in FM (Deitos et al., 2015), whereas in MDD there is a vast literature showing lower serum BDNF (Karege et al., 2002). Thus, these results suggest that this neurotrophic factor could be a correlate marker of distinct mechanisms that underpin the pathophysiology of FM and MDD. The BDNF is secreted by the microglia and it participates in the adaptative and protective neuroplasticity processes. However, in chronic pain, this mechanism is likely to be overactivated and raise a counterproductive response, in the sense that the microglia-to-neuron communication might attenuate the pain inhibitory action of GABA and the glycine receptor-mediated inhibition (Ferrini and De Koninck, 2013). This hypothesis is supported by compelling evidence that BDNF is a ubiquitous pain mediator at many levels of the nervous system. Given this, it would be hard to conclude that the generation of BDNF is a compensatory mechanism specific to chronic pain conditions (i.e., FM, chronic inflammatory, and neuropathic pain). Although in the current study we have not observed a significant relationship between the BDNF adjusted index and the inhibitory function of motor cortex indexed by the SICI, this may be explained by an error type II, since other studies found a significant correlation between the SICI and BDNF. Indeed, the adjusted index of BDNF was used as a measure to summarize several factors associated with the BDNF secretion (i.e., antidepressant, anticonvulsants, age, etc.). Thereby, we need parsimony in the interpretation of this inter-relationship, since this study is an exploratory and approximately 65% of FM presented psychiatric diagnosis and used psychotropic medications (tricyclic antidepressant, pregabalin, etc.). Thereby, it is possible that intermediates confounding factors did not have entirely controlled (Cole and Hernán, 2002) or a non-significant *p*-value after adjustment reflects the absence of a relevant effect these relationships in this sample (Baguley, 2004).

Also, we identified a more substantial dysfunction in DPMS in FM compared to MDD, and the BDNF adjusted index was positively correlated with the disinhibition of DPMS. This result is aligned with an earlier study that found similar results related to the increase of serum BDNF and the disruption of the inhibitory function of DPMS in chronic musculoskeletal pain (Botelho et al., 2016; Caumo et al., 2016). Likewise, it has been demonstrated that the increased synthesis of BDNF in the nociceptive pathways is responsible for increasing neuronal excitability by causing disinhibition in dorsal horn neurons in the spinal cord (Ferrini and De Koninck, 2013). In the brain, the BDNF has been shown to activate descending nociceptive facilitation in the nucleus raphe magnus (Zhang et al., 2013). Also, at the periaqueductal gray neurons, the BDNF has a central role for orchestrating descending antinociception (Lewis et al., 2012; Nijs et al., 2014). Thus, the disinhibition of the motor cortex indexed by SICI together with the dysfunction of the descending antinociceptive mechanisms is an essential feature of FM, which we did not observe in depression. However, it is difficult to determine whether the deterioration of cortical inhibition, changes in BDNF and the dysfunction of DPMS may be an underlying pathophysiological mechanism of the disease or a disease severity state-dependent phenomenon.

Although our results are likely to help to advance in the comprehension of changes in measures related to neuroplasticity in the two disorders, our results are correlational and do not allow a causality relationship. This study has some limitations: Firstly, TMS consists of an indirect neurophysiological measure intended to assess the activity of a neurotransmitter system. Second, psychiatric disorders remain a potential confounding factor, and they cannot have been adequately controlled, even if anxiety, depression, catastrophizing pain behavior, and psychiatric diagnosis were assessed. More than 70% (12/17) of FM group suffered from any mental illnesses. However, this finding is expected, as the emotional burden is a recurrent finding in chronic pain syndromes. Third, we must address the effect of psychotropic medicines under cortical excitability because the regular prescription of these medicines deliberates the proper treatment of both disorders. Nevertheless, it is critical to mention that different changes in cortical excitability produced using psychotropic medications might produce distinctive outcomes in acute and long-term use. Fourth, we performed this study only in females, and it is essential to consider a sex effect in pain perception and modulation. Likewise, our results must be carefully interpreted, given the design of this study. Further, research designed to address differences and similarities between FM and MDD are required to claim if the neuroplastic and neurophysiological measures constitute differential biomarkers of their pathophysiological mechanisms.

## Conclusion

In conclusion, these findings support the hypothesis that in FM a deteriorated function of cortical inhibition, indexed by a higher SICI parameter, and a lower function of the DPMS, together with higher levels of BDNF indicate that FM has different pathological substrates from depression. They suggest that an up-regulation phenomenon of intracortical inhibitory networks associated with a disruption of the DPMS function occurs in FM.

## Ethics Statement

This study has been performed in accordance with the ethical standards as laid down in the 1964 Declaration of Helsinki and its later amendments. The protocol was approved by the Ethics Committee Board of the Clínicas Hospital de Porto Alegre (Institutional Review Board IRB 0000921). All individuals provided oral and written consent before their engagement in the study.

## Author Contributions

All authors listed have made a substantial, direct and intellectual contribution to the work, and approved it for publication.

## Conflict of Interest Statement

The authors declare that the research was conducted in the absence of any commercial or financial relationships that could be construed as a potential conflict of interest.
